# Angiogenic Factor AGGF1 Promotes Therapeutic Angiogenesis in a Mouse Limb Ischemia Model

**DOI:** 10.1371/journal.pone.0046998

**Published:** 2012-10-23

**Authors:** Qiulun Lu, Yihong Yao, Yufeng Yao, Shizhi Liu, Yuan Huang, Shan Lu, Ying Bai, Bisheng Zhou, Yan Xu, Lei Li, Nan Wang, Li Wang, Jie Zhang, Xiang Cheng, Gangjian Qin, Wei Ma, Chengqi Xu, Xin Tu, Qing Wang

**Affiliations:** 1 Key Laboratory of Molecular Biophysics of the Ministry of Education, College of Life Science and Technology, Center for Human Genome Research, Cardio-X Institute, Huazhong University of Science and Technology, Wuhan, People’s Republic of China; 2 Institute of Cardiology, Union Hospital, Tongji Medical College, Huazhong University of Science and Technology, Wuhan, People’s Republic of China; 3 Feinberg Cardiovascular Research Institute, Northwestern University Feinberg School of Medicine, Chicago, Illinois, United States of America; 4 The First Hospital of Wuhan City, Wuhan, People’s Republic of China; 5 Center for Cardiovascular Genetics, Department of Molecular Cardiology, Lerner Research Institute, Cleveland Clinic, Cleveland, Ohio, United States of America; University of Otago, New Zealand

## Abstract

**Background:**

Peripheral arterial disease (PAD) is a common disease accounting for about 12% of the adult population, and causes significant morbidity and mortality. Therapeutic angiogenesis using angiogenic factors has been considered to be a potential treatment option for PAD patients. In this study, we assessed the potential of a new angiogenic factor AGGF1 for therapeutic angiogenesis in a critical limb ischemia model in mice for PAD.

**Methods and Results:**

We generated a unilateral hindlimb ischemia model in mice by ligation of the right common iliac artery and femoral artery. Ischemic mice with intrasmuscular administration of DNA for an expression plasmid for human *AGGF1* (*AGGF1* group) resulted in increased expression of both *AGGF1* mRNA and protein after the administration compared with control mice with injection of the empty vector (control group). Color PW Doppler echocardiography showed that the blood flow in ischemic hindlimbs was significantly increased in the *AGGF1* group compared to control mice at time points of 7, 14, and 28 days after DNA administration (n = 9/group, *P* = 0.049, 0.001, and 0.001, respectively). Increased blood flow in the *AGGF1* group was correlated to increased density of CD31-positive vessels and decreased necrosis in muscle tissues injected with *AGGF1* DNA compared with the control tissue injected with the empty vector. Ambulatory impairment was significantly reduced in the *AGGF1* group compared to the control group (*P* = 0.004). The effect of AGGF1 was dose-dependent. At day 28 after gene transfer, *AGGF1* was significantly better in increasing blood flow than *FGF-2* (*P* = 0.034), although no difference was found for tissue necrosis and ambulatory impairment.

**Conclusions:**

These data establish *AGGF1* as a candidate therapeutic agent for therapeutic angiogenesis to treat PAD.

## Introduction

Peripheral arterial disease (PAD) is caused by atherosclerosis, which results in progressive narrowing and occlusion of the peripheral arteries and inhibits blood flow to the lower extremities [1], [2]. The prevalence of PAD is increasing in the modern aging society, and reaches about 12% of the adult population [3], [4]. In the US, approximately 8 to 12 million people are affected with PAD [5]. In Germany, the prevalence of PAD for women and men aged ≥65 years was 17% and 20%, respectively [6].

The clinical presentation of PAD may vary from being asymptomatic to presenting with a serious symptom of intermittent claudication [1], [7]. In severe cases, PAD significantly affects quality of life, and increases morbidity and mortality. Approximately 25% patients with critical limb ischemia die in one year [8]. Moreover, in patients with PAD the prevalence of coronary artery disease (CAD) is about 46% to 71% [1], [9], [10], and at least 10% of them suffered cerebrovascular disease [1], [2], [11], [12].

The current treatment for PAD focuses on decreasing cardiovascular and cerebrovascular morbidity and mortality and on relieving PAD symptoms [13]. Pharmacological treatments with lipid-lowering, antihypertensive, and antithrombotic medication are used to prevent myocardial infarctions (MIs) and strokes [1], [13]. Some PAD patients may require leg amputation to relieve the unbearable pain and life-threatening situation. To avoid amputation or other serious prognosis, angioplasty, stenting and peripheral artery bypass surgeries are used to restore blood flow to the legs in some patients [1], [13], [14]. However, PAD patients undergoing vascular surgeries had a significantly worse long-term prognosis than CAD patients with similar procedures [15]. Furthermore, many PAD patients are not suitable candidates for interventional or revascularization surgeries. Therefore, therapeutic angiogenesis, i.e. promotion of angiogenesis and microcirculation using angiogenic factors, has been proposed as a new and potential treatment strategy for PAD patients in the last decade [16]. The administration of angiogenic factors, either as naked plasmid DNA or recombinant proteins, may promote neovascularization, augment the collateral circulation, and enhance blood perfusion to ischemic tissues [16], [17]. Therapeutic angiogenesis for PAD made some progress, but also met some problems [16], [18]. Several angiogenic factors, for example, vascular endothelial growth factor A (VEGF), fibroblast growth factors (FGFs), hepatocyte growth factor (HGF), and platelet-derived growth factor (PDGF), have been tested to treat critical limb ischemia in PAD patients or in animal models. These studies provided encouraging results, however, therapeutic angiogenesis for PAD is considered to be still at its infancy and adverse effects in some cases occurred, including vascular leakage, transient edema, and hypotension with administration of *VEGF* and *FGFs* [19], [20]. In addition, the efficacy of therapeutic angiogenesis may be limited by the few number of angiogenic factors available for selection. Therefore, it is crucial that new angiogenic factors should be identified and tested for treating PAD.

*AGGF1* is a new angiogenic factor identified by our group through genetic analysis of a congenital vascular disorder called Klippel-Trenaunay syndrome (KTS) [21], [22]. AGGF1 protein contains several functional domains, including a coiled-coil motif at the N-terminus, an OCtamer REpeat (OCRE) domain, a Forkhead-associated (FHA) domain, and a G-patch domain at the C-terminus. The specific functions of these domains are unknown. We have demonstrated that AGGF1 promotes angiogenesis as potentially as VEGF in a chicken embryo angiogenesis assay [21]. Recently, we reported that *GATA1* could regulate the expression of *AGGF1* through two *GATA1* binding sites in the promoter region of *AGGF1* [23]. Knockdown of *GATA1* expression reduced *AGGF1* expression, which resulted in reduced endothelial capillary vessel formation in a matrigel vessel tube formation assay, and purified recombinant AGGF1 protein can rescue the defect when added in the media [23]. The angiogenic potential of *AGGF1* prompted us to investigate whether therapeutic angiogenesis with administration of naked DNA for an *AGGF1* expression construct is capable of increasing blood flow in a mouse hindlimb ischemia model.

## Results

### Successful Expression of *AGGF1* by Administration of Naked Plasmid DNA into Gastrocnemius Muscle

**Figure 1:**
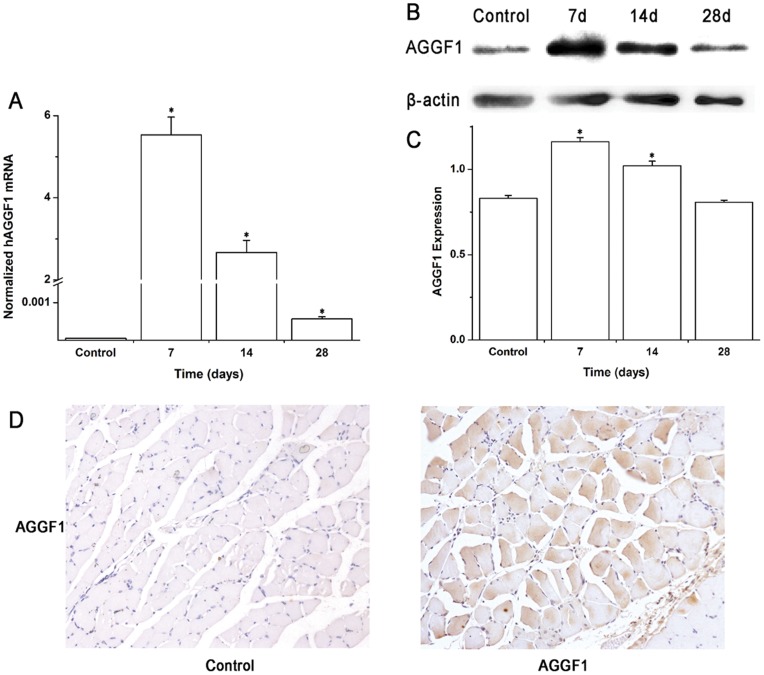
Successful overexpression of *AGGF1* mRNA and protein in skeletal muscle by gene transfer using a plasmid-based delivery system. **A)** Real time PCR analysis for expression of human *AGGF1* mRNA derived from an *AGGF1* expression plasmid injected into the gastrocnemius muscle in a mouse ischemic hindlimb model. The RT-PCR primers are specific to human *AGGF1* and not able to amplify the endogenous mouse *AGGF1* mRNA. Time points are in days after the injection of plasmid DNA. Injection of the empty vector DNA served as negative control. The data are shown as the amount of plasmid-derived human *AGGF1* mRNA normalized to control *GAPDH* levels. To avoid the contamination of plasmid DNA, RNA samples were treated with DNase. In addition, RT-PCR analysis with RNA samples without reverse transcription did not yield any product. **B**) Expression of AGGF1 protein in gastrocnemius muscles by Western blot analysis at time points of 7, 14, 28 days after plasmid DNA was injected. The anti-AGGF1 antibody recognizes both human AGGF1 derived from the injected plasmid and endogenous mouse AGGF1 protein. **C**) The images from Western blot analysis in **B**) were scanned, quantified, and plotted. The intensity of AGGF1 signal was normalized to the signal of control β-actin. **D**) Immunohistochemical analysis of the sections of ischemic hindlimb muscle from mice injected with an empty vector (Control) and with an *AGGF1* expression plasmid (AGGF1) with an anti-AGGF1 antibody seven days later by injection of plasmid DNA. *, *P*<0.05.

Previous reports by others demonstrated that the efficiency of gene transfer by direct injection of DNA for an expression plasmid was determined by the amount of plasmid DNA and the injection volume [24]. An increase in injection volume and one site injection rather than separate injections at multiple sites resulted in higher transfection efficiencies. Therefore, we injected 0.2 mg of purified plasmid DNA in a 0.15 ml of volume for pcDNA3.1-AGGF1-FLAG (an expression construct for *AGGF1*) or control vector pcDNA3.1-FLAG into one site in the gastrocnemius muscle. At different time points, muscle tissue samples from ischemic and non-ischemic hindlimbs were harvested for real-time RT-PCR and Western blot analyses to measure the expression levels of *AGGF1*. As shown in Figure 1A, human *AGGF1* mRNA in ischemic hindlimb tissues from mice injected with pcDNA3.1-AGGF1-FLAG increased dramatically compared with that from mice injected with control vector pcDNA3.1-FLAG one week after the injection of DNA (*P*<0.001). The expression of human *AGGF1* remained high for two weeks after the injection, but decreased to a lower level four weeks after the injection (Figure 1A).

Similar results were obtained with Western blot analysis (Figure 1B). For Western blot analysis, the anti-AGGF1 antibody can also detect the endogenous mouse AGGF1 protein in the mice injected with control vector pcDNA3.1-FLAG. Moreover, increased human AGGF1 protein expression was found at the time points of seven days and fourteen days, but not at twenty-eight days in mice injected with pcDNA3.1-AGGF1-FLAG (Figure 1B). Consistent with these results, immunohistochemistry analysis also showed higher AGGF1 protein expression in tissues from mice injected with pcDNA3.1-AGGF1-FLAG than that from mice injected control vector pcDNA3.1-FLAG one week after the DNA injection (Figure 1D).

Together, these data suggest that direct injection of naked plasmid DNA for an *AGGF1* expression construct into gastrocnemius muscles results in a successful high level of expression of both *AGGF1* mRNA and protein.

### Increased *AGGF1* Expression Stimulated an Increase of Blood Flow in the Ischemic Hindlimb

**Figure 2:**
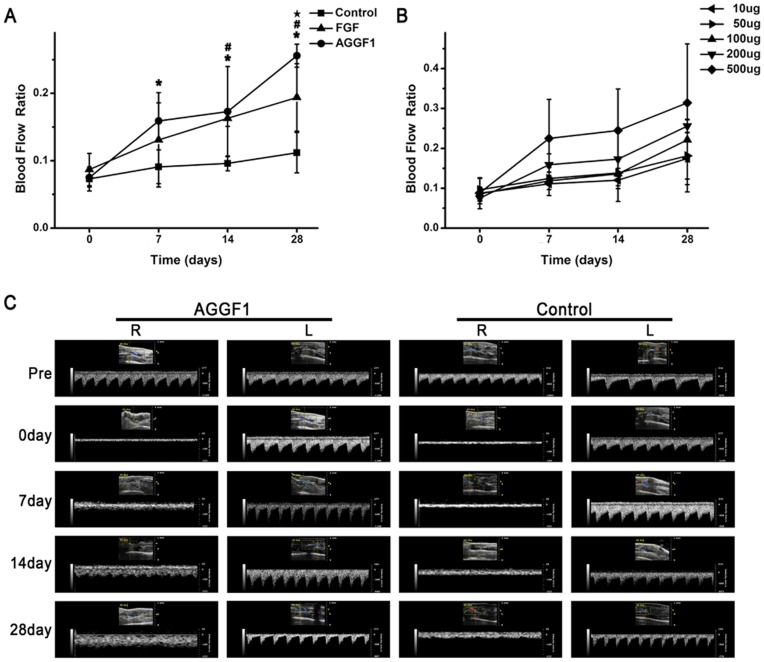
Effects of gene transfer for human *AGGF1* by intramuscular administration of plasmid DNA on blood flow in a mouse ischemic hindlimb model. **A**) Injection of *AGGF1* plasmid DNA increased blood flow compared to injection of DNA for an empty vector (control). *FGF-2* plasmid DNA was used as a positive control. The right femoral artery was ligated to mimic unilateral hindlimb ischemia. One group of ischemic mice received DNA for a human *AGGF1* expression plasmid, and the other group received DNA for a control empty plasmid one day after the ligation surgery. The ratio of blood flow in the ischemic leg over that in the nonischemic limb at the indicated time points (in days) was plotted. The blood flow was measured by high resolution microultrasound. **B**) Dose-response curve of blood flow for varying amounts of *AGGF1* plasmid DNA. **C**) Representative images from micro-ultrasound. R, ischemic leg undergone surgery and ligation; L, leg undergone mock surgery. *, *P*<0.05 for comparing *AGGF1* vs control at the same time point; #, *P*<0.05 for comparing *FGF2* vs control at the same time point; ★, *P*<0.05 for comparing *AGGF1* vs FGF2 at the same time point.

We measured the change of blood flow in the hindlimb ischemia model using a high-resolution micro-ultrasound system. One day after surgery (ligation of arteries), the ratio of blood flow in the ischemic leg over that in the non-ischemic leg decreased sharply to 10% of the level before the surgery in mice injected with either pcDNA3.1-AGGF1-FLAG or control pcDNA3.1-FLAG (Figure 2A, 2C). In addition, the wave form of blood flow also changed dramatically and it was difficult to identify the pulse of blood flow after the surgery.

After the ischemia was confirmed by micro-ultrasound, 0.15 ml of PBS containing 0.2 mg of purified DNA was injected into the gastrocnemius muscle close to the ligation site of the ischemic hindlimb. In mice injected with pcDNA3.1-AGGF1-FLAG, the ratio of blood flow of ischemic/nonischemic hindlimbs increased significantly one week after DNA injection (Figure 2A, 2C) compared to control mice injected with pcDNA3.1-FLAG (*P*<0.05, n = 9). The increased blood flow in mice injected with pcDNA3.1-AGGF1-FLAG remained at the time point of two weeks. Four weeks after the injection of DNA, the blood flow was approximately 2.29 fold higher in mice injected with pcDNA3.1-AGGF1-FLAG than those injected with pcDNA3.1-FLAG (0.256±0.017 vs. 0.112±0.03; *P* = 0.001, n = 9).

We compared the effect of *AGGF1* on blood flow to *FGF-2*, a potent angiogenic factor. The same amount of DNA for *AGGF1* and *FGF-2* expression plasmids (200 µg) was administered. Similar to *AGGF1*, *FGF-2* also strongly stimulated blood flow in mouse hindlimb ischemic models. No difference was observed 7 and 14 days after gene transfer between the *AGGF1* group and *FGF-2* group. However, at day 28, *AGGF1* performed significantly better than *FGF-2* in stimulating blood flow in mouse hindlimb ischemic models (*P* = 0.034).

We then analyze the effect of different amounts of *AGGF1* expression plasmid DNA on blood flow. As the amount of *AGGF1* DNA increased from 10 µg to 500 µg, the blood flow increased (Figure 2B).

**Figure 3:**
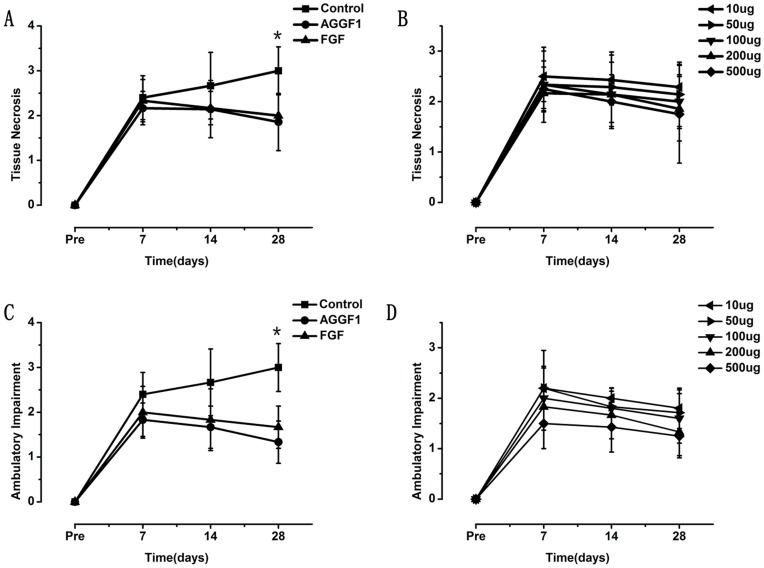
Effects of gene transfer for human *AGGF1* by intramuscular administration of plasmid DNA on tissue necrosis and ambulatory impairment in a mouse ischemic hindlimb model. **A**) Effect of overexpression of human *AGGF1* on tissue necrosis. *FGF-2* plasmid DNA was used as a positive control. Empty vector DNA was used as negative control (control). **B**) Dose-response curve of tissue necrosis with varying amounts of *AGGF1* plasmid DNA. **C**) Effect of overexpression of human *AGGF1* on ambulatory impairment. *FGF-2* plasmid DNA was used as a positive control. Empty vector DNA was used as negative control (control). The scores of ambulatory impairment reflected the functional recovery of hindlimb ischemia after surgeries. **D**) Dose-response curve of ambulatory impairment with varying amounts of *AGGF1* plasmid DNA. Data are shown as mean ±SEM (n = 9 mice for each group). *, *P*<0.05.

We also evaluated ischemic limb functions by scoring limb tissue necrosis and ambulatory impairment. Both scores decreased markedly in mice injected with pcDNA3.1-AGGF1-FLAG compared with those injected with pcDNA3.1-FLAG (tissue necrosis score in Figure 3A and 3B and ambulatory impairment score in Figure 3C and 3D). When compared to the same amount of *FGF-2* expression plasmid DNA, *AGGF1* was as equally competent as *FGF-2* in inhibiting tissue necrosis and ambulatory impairment (Figure 3A and 3C). As the amount of *AGGF1* DNA increased from 10 µg to 500 µg, both the tissue necrosis score and ambulatory impairment were reduced (Figure 3B and 3D).

### Increased *AGGF1* Expression Inhibited Necrosis in the Ischemic Hindlimb

**Figure 4:**
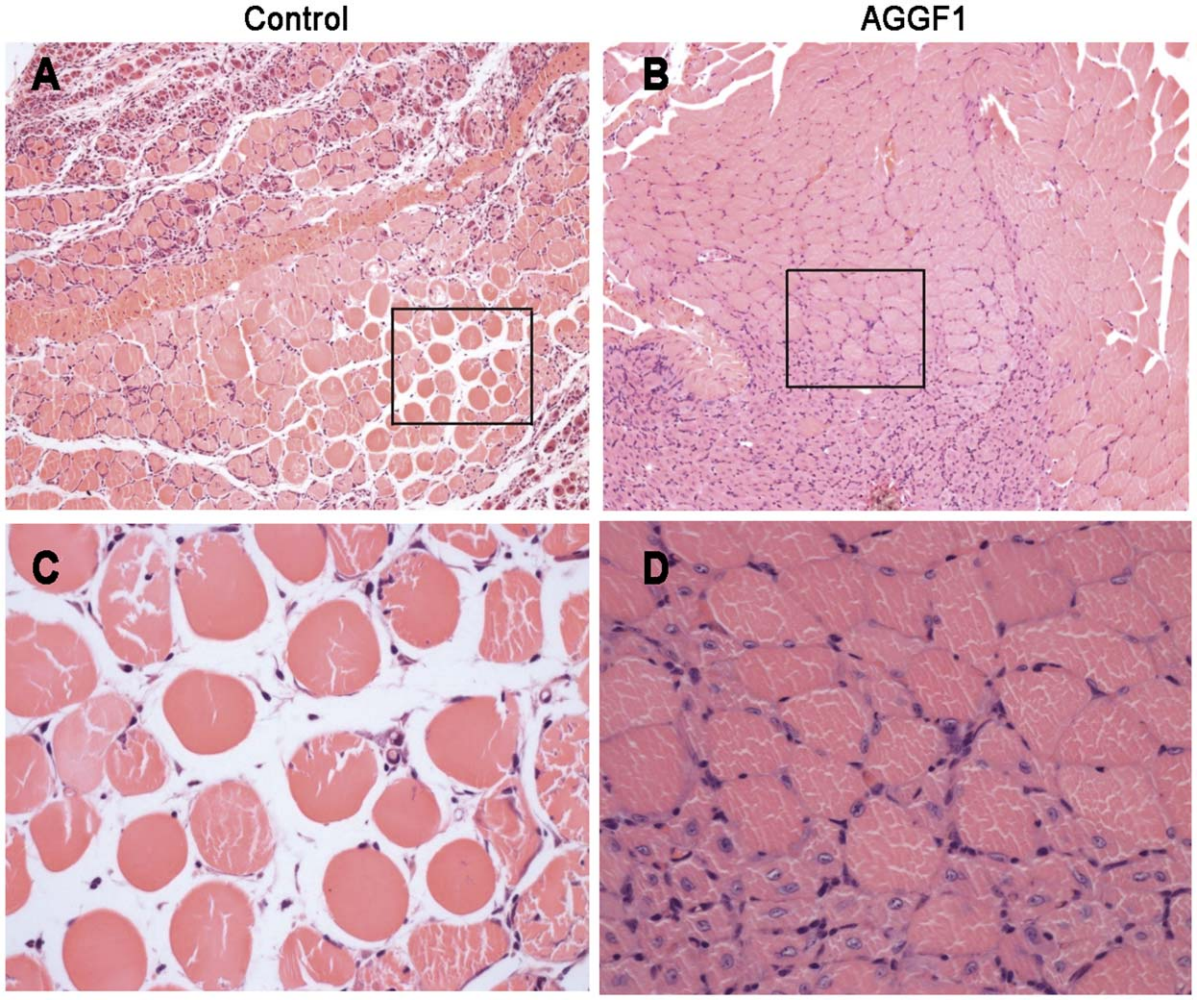
Histological examinations of muscle tissues. A ) Ischemic hindlimb muscle sections from ischemic mice showed severe necrosis 7 days after injection of empty vector DNA. **B**) Ischemic mice injected with *AGGF1* plasmid DNA showed much less necrosis and retained intact muscle 7 days after injection of DNA. **C**) Enlarged image for a section from **A**). **D**) Enlarged image for a section from **B**).

H&E histological staining was carried out for ischemic hindlimb muscle sections for both pcDNA3.1-AGGF1-FLAG and control pcDNA3.1-FLAG groups of mice 7 days after DNA injection. In the control mice, ischemic hindlimb muscle sections showed centralization of nuclei and many necrosed muscle fibers (Figure 4), which indicated severe necrosis. However, in mice injected with pcDNA3.1-AGGF1-FLAG, centralized nuclei were less prominent, and the number of necrosed muscle fibers was highly reduced (Figure 4). Overall, ischemic gastrocnemius muscle sections from mice with overexpression of *AGGF1* showed less gross tissue necrosis than that from mice without *AGGF1* overexpression.

### Increased *AGGF1* Expression Induced Angiogenesis in the Ischemic Hindlimb

**Figure 5:**
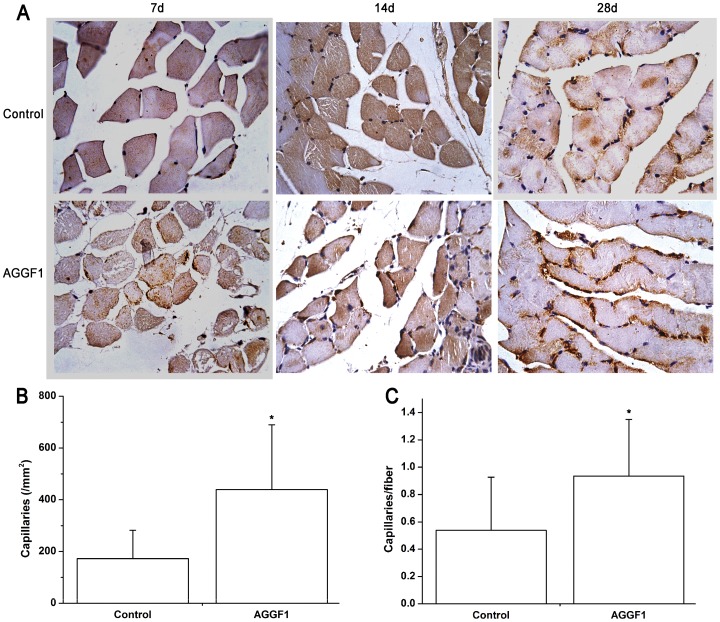
Overexpression of human *AGGF1* stimulated angiogenesis in a mouse hindlimb ischemia model. A ) Representative images of sections of ischemic hindlimb muscles immunostained with an anti-CD31 antibody 7, 14, or 28 days after injection of *AGGF1* plasmid DNA or empty vector DNA (control). **B**) Quantification of density of CD-31-positive vessels per mm^2^ in ischemic muscles 28 days after injection of DNA. **C**) The number of CD-31-positive vessels per muscular fiber 28 days after injection of DNA. *, P<0.05.

The effect of overexpression of human *AGGF1* on angiogenesis was examined in the ischemic hindlimbs by immunostaining of gastrocnemius muscle sections with an anti-CD31 antibody four weeks after DNA injection (Figure 5A). The density of CD31-positive vessels was significantly higher in mice injected with pcDNA3.1-AGGF1-FLAG than control pcDNA3.1-FLAG group of mice (0.934±0.413/mm^2^ vs. 0.538±0.388/mm^2^, *P*<0.01) (Figure 5B). In addition, the number of CD31-positive vessels in fibers was also markedly increased in mice injected with pcDNA3.1-AGGF1-FLAG than the control group of mice with pcDNA3.1-FLAG injection (439.2±250.1 vs. 172.5±109.4, *P*<0.05) (Figure 5C). These data suggest that overexpression of human *AGGF1* induces angiogenesis in ischemic gastrocnemius muscles.

**Figure 6:**
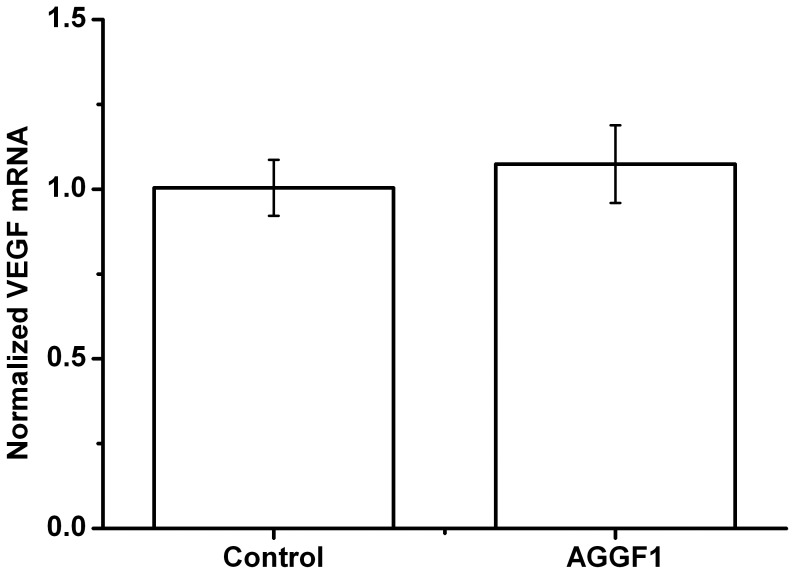
Overexpression of human *AGGF1* in a mouse hindlimb ischemia model does not affect the expression level of *VEGF*. Seven days after delivery of *AGGF1* expression plasmid DNA (empty vector as control), mice were sacrificed and gastrocnemius muscle tissues were excised and used for isolation of total RNA and follow-up real time RT-PCR analysis of the mouse *VEGF* gene. Note that *VEGF* expression did not show any difference between *AGGF1* and control (*P*>0.05).

To exclude the possibility that increased angiogenesis in gastrocnemius muscle sections was related to *VEGF*, we carried out real time RT-PCR analysis for mouse *VEGF* using RNA samples isolated from gastrocnemius muscle tissues. No significant difference was found for the expression level of *VEGF* in tissues injected with *AGGF1* expression plasmid DNA and with an empty vector DNA (Figure 6).

## Discussion

The data in the present study indicate that gene transfer by administration of plasmid DNA for an *AGGF1* expression construct promotes blood flow and improves hindlimb muscle microcirculation in a mouse model of PAD induced by ligation of both common iliac artery and femoral artery (Figure 2). Further studies showed that increased blood flow in the ischemic limb correlated with increased density of CD31-positive vessels and reduced tissue necrosis (Figures 3, 4, 5).

Critical limb ischemia is the most advanced stage of PAD, and is always associated with severe atherosclerosis. Atherosclerosis in peripheral arteries decreases blood flow and oxygen supply to the muscle tissue of limbs and toes, which may result in tissue necrosis due to hypoxia. This study is the first to investigate the potential of *AGGF1* as a new strategy for therapeutic angiogenesis for critical limb ischemia. Our data indicate that *AGGF1* can serve as a novel therapeutic agent for the treatment of critical limb ischemia as increased *AGGF1* expression in skeletal muscles enhanced limb function, increased blood flow, and healed ischemic ulcers in a mouse hindlimb ischemia model.

For gene transfer involved in therapeutic angiogenesis, several delivery methods are used. Administration of purified recombinant proteins achieved some positive results, but short half-life of proteins and long-term toxicity with repeated injections may cause system toxicity. Infection with adenoviruses with the target gene is effective in achieving stable and long-term expression, but may induce adverse immune reactions. Davis et al [25] reported that injection of naked plasmid DNA appeared to be better for overexpressing a target gene in skeletal muscles than viral vectors because skeletal muscle cells were able to take plasmid DNA up. Thus, direct injection of plasmid DNA into skeletal muscle tissues has become an alternative to achieve a high level of gene transfer to avoid significant disadvantages with injection of a protein and an adenoviral vector. The data in the present study again showed that direct injection of DNA for an *AGGF1* expression plasmid successfully overexpressed *AGGF1* in the ischemic tissues (Figure 1). Two recent clinical trials, HGF-STAT and TALISMAN201 utilized a plasmid-based angiogenic gene delivery system for HGF and FGF-1, respectively, and achieved some encouraging results (improved transcutaneous oxygen measurements TcPO2 in the HGF case and a reduced risk of major amputation and death in the FGF-1 case) [26], [27]. More clinical trials are needed to replicate these findings. Similarly, future clinical studies for therapeutic angiogenesis using *AGGF1* with a plasmid-based gene delivery system are needed to unequivocally establish the efficacy of *AGGF1* treatment for PAD.

Tsurumi et al showed that administration of naked plasmid DNA encoding *VEGF* increased regional blood flow to the transfected thigh muscle and distal lower limb muscle by 1.5-fold in a rabbit ischemic hindlimb model [28]. Hiraoka et al. reported that in a rat model of hindlimb ischemia, injection of *VEGF* plasmid DNA increased blood flow by about 30% [24]. Taniyama et al. showed that injection of naked *HGF* plasmid DNA into skeletal muscle resulted in a 70% increase in blood flow in a rat model of hindlimb ischemia three weeks after the injection [29]. We demonstrated that injection of naked plasmid DNA for an *AGGF1* expression construct resulted in a 2.29-fold increase in blood flow (Figure 2). Furthermore, our parallel comparison analysis found that *AGGF1* was significantly better than *FGF-2* in stimulating blood flow 28 days after gene transfer (*P* = 0.034), although no significant difference was found for day 7 and day 14 (Figure 2). Therefore, *AGGF1* appears to be an excellent choice for therapeutic angiogenesis for critical limb ischemia.

Some side effects were uncovered in previous studies involving therapeutic angiogensis for treating limb ischemia in PAD using VEGF and FGFs. The major adverse effects include increased vascular permeability (resulting in vascular leakage) and transient edema [19], [20]. In contrast to VEGF, AGGF1 is required for maintaining the vascular integrity because adult heterozygous *AGGF1^+/−^* knockout mice showed increased vascular permeability in an assay using Evan’s blue dye [30]. During the AGGF1 treatment in a hindlimb ischemic mouse model for PAD, we did not observe any edema. However, future studies are needed to determine whether a larger dose of *AGGF1* DNA injection may result in side effects of edema or other undesirable abnormalities in major organs such as the heart, livers, kidneys, lungs and other organs.

A long-standing question about the mode of action of AGGF1 during angiogenesis is whether it acts by an autocrine or paracrine mechanism [21], [31]. Because the AGGF1 protein is expressed and secreted by endothelial cells, we suggested that AGGF1 may act by an autocrine mode, however, we also stated that a paracrine mode of action was possible because AGGF1 was highly expressed in vascular smooth muscle cells [21]. In the present study we show that overexpression of *AGGF1* in skeletal muscle cells can apparently promote strong angiogenesis (Figure 5). The data strongly suggest that AGGF1 is able to act by a paracrine mechanism. The molecular mechanism by which AGGF1 promotes angiogenesis remains to be further explored. Decreased expression of *AGGF1* resulted in massive apoptosis of endothelial cells, whereas recombinant AGGF1 can rescue this abnormality when coated onto wells of culture plates [23]. Similar results were obtained for endothelial cell migration [23]. Thus, AGGF1 promotes angiogenesis by acting as a surviving factor for endothelial cells or by promoting endothelial cell migration. We have shown that AGGF1 interacts with TWEAK [21], which promotes angiogenesis as potently as VEGF and FGF-2 [32]. TWEAK acts by binding to its receptor fibroblast-growth factor inducible 14 (Fn14). Thus, AGGF1 may promote angiogenesis by interacting with TWEAK. Alternatively, AGGF1 may have its own receptor during angiogenesis. Future studies can focus on identifying the receptor for AGGF1 and the detailed molecular signaling mechanism involved in AGGF1-mediated angiogenesis.

In conclusion, the data in this study suggest that gene transfer using *AGGF1* naked plasmid DNA significantly increases blood flow by promoting angiogenesis and inhibiting tissue necrosis in a mouse model of critical hindlimb ischemia for PAD. Therapeutic angiogenesis with *AGGF1* may be beneficial to patients with not only PAD, but also other ischemic conditions such as ischemic heart disease, MIs, and strokes.

## Materials and Methods

### Animals and a Hindlimb Ischemic Model

Male C57BL/6 mice were obtained from the Animal Facility at Wuhan University Zhong Nan Hospital. Animal care and experimental procedures were approved by the Ethics Committee of College of Life Science and Technology, Huazhong University of Science and Technology.

Male mice at the age of 10–12 weeks were used. The hindlimb ischemic model was created as described previously [33], [34]. The mice were anesthetized with an intraperitoneal injection of sodium pentobarbital (50 mg/kg). After incision of the skin in the inguinal region, the femoral artery was separated from the femoral vein and nerve. Both common iliac artery and femoral artery in the right side were tightly ligated with 6/0 Ethilon sutures. Subsequently, the skin incision was closed by sutures. The arteries at the left side were not ligated and served as control.

### Gene Transfer

The cDNA for human *AGGF1* was obtained using RT-PCR analysis with total RNA samples from HeLa cells (ATCC). The PCR product was digested with BamH I and Not I designed into the PCR primers, and cloned into vector pcDNA3.1-FLAG, resulting in a mammalian expression construct for *AGGF1* (pcDNA3.1-AGGF1-FLAG). The entire insert in the *AGGF1* expression construct was sequenced and verified. The expression plasmid for *FGF-2* was developed as for *AGGF1*.

One day after the ischemia surgery, 0.15 ml of 1.33 µg/µl DNA (200 µg) for either pcDNA3.1-AGGF1-FLAG or pcDNA3.1-FLAG was injected into the gastrocnemius muscle close to the ligation site of the right ischemic hindlimb [24], [35], [36]. Mice were sacrificed at various time points after the injection and characterized.

### Blood Flow Analysis

The blood flow in both legs was measured using a Vevo 2100 High-Resolution Microultrasound System (Visualsonics Inc, Toronto, Canada) immediately before the ischemic surgery and at different time points of postoperative days 1, 8, 15, 22 and 29. The Microultrasound System allows for repeated, noninvasive and quantitative measurements of the blood flow of the certain sites of the artery. The surgical outcome of limb ischemia at the right leg, but not the left leg, was confirmed by the Microultrasound System. The mice were anesthetized with 1% isoflurane, placed on a heat pad, and kept at 37°C to minimize data variations caused by fluctuating body temperatures. Excess hairs were removed by depilatory cream from the urite to the limb before analysis [37].

### Physical Examinations of Tissue Necrosis and Ambulatory Impairment

Tissue necrosis was scored as described [38]. “0” was for no necrosis, “1” for necrosis of one toe, “2” for necrosis of two or more toes “3” for necrosis of the foot, “4” for necrosis of the leg, and “5” for autoamputation of the entire leg.

The severity of ambulatory impairment was assessed by using the following scale as described [38], [39]. “0” was for normal response (plantar/toe flexion in response to tail traction), “1” for plantar but not toe flexion, “2” for no plantar or toe flexion, “3” for dragging of foot, and “4” for spontaneous movement of non-ischemic hindlimb. Physical examinations of both tissue necrosis and ambulatory impairment were performed by an observer who was blinded to treatments.

### Quantitative Real-time PCR Analysis

Total RNA samples were extracted from gastrocnemius muscles using TRIzol (Invitrogene), precipitated with isopropanol, and treated with RQ1 RNase-Free DNase (Promega) to eliminate DNA contamination. The synthesis of the first-strand cDNA was performed using M-MLV Reverse Transcriptase (Promega). Real-time RT-PCR analysis was performed using the FastStart Universal SYBR Green Master (Roch) and a 7900 HT Fast Real-Time PCR System (ABI).

The sequences for RT-PCR primers are 5′- GATCAAGGCAGTCAAAATGGCA-3′ (sense) and 5′- CCTCCTTACTTAGTGTTGGACCA-3′ (antisense) for AGGF1, 5′- AGGTCGGTGTGAACGGATTTG-3′ (sense) and 5′- TGTAGACCATGTAGTTGAGGTCA-3′ (antisense) for glyceraldehyde 3-phosphate dehydrogenase (GAPDH), and 5′- TGCACCCACGACAGAAGGAGAGC-3′ (sense) and 5′- CGGCACACAGGACGGCTTGAAG-3′ (antisense) for mouse VEGFA. GAPDH served as an internal standard. The data were analyzed using 2^−△△Ct^ relative expression quantity as described [40].

### Western Blotting Analysis

Ischemic and non-ischemic calf muscles were weighed, homogenized, and centrifuged in RIPA buffer containing protease and phosphatase inhibitor cocktails (Roche Diagnostics). A 20 µg of total protein extracts was separated on 10% SDS-PAGE gels, and transferred to nitrocellulose membranes (Millipore). The membranes were blocked with 4% milk powder, washed with TBST, and incubated with a polyclonal anti-AGGF1 antibody or a control anti-β-actin antibody (Proteintech) overnight at 4°C. The membranes were washed with TBST and incubated with peroxidase-conjugated anti-rabbit IgG (Sigma) as a secondary antibody. The membranes were washed with TBST, and developed using Supersignal Chemiluminescence Substrate system (Pierce). The protein signal was imaged and analyzed using ChemiDoc XRS (BioRad).

### Histology and Immunohistochemistry Analysis

Immunohistochemistry was performed as described [41]. Muscle samples were fixed in 4% paraformaldehyde, paraffin embedded, and sectioned (8 µm). The sections were deparaffinized using xylene (dimethyl benzene), and heated in citrate buffer (pH 6.0) or 1 mmol/L EDTA (pH 8.0) to retrieve antigen. Slides were then blocked with 3% H_2_O_2_ and then 10% goat serum, and incubated with the primary anti-AGGF1 antibody or an anti-CD31 antibody for 1 hour at room temperature for overnight at 4°C. The sections were then washed with PBS and incubated with a secondary, biotinylated anti-rabbit IgG for 15 minutes, followed by washing in PBS, and incubation with SABC kit (Boster). The signal was developed with a DAB chromogen kit (Dako), and counterstained with hematoxylin. Replacement of the primary antibody with PBS served as a negative control. All measurements were performed in a blinded manner. Six sections were randomly studied for each mouse and viewed at 40X viewing fields to measure average positive immunoactivity. To avoid confounding caused from trauma effects of the needle injection, the analysis was made outside of the needle injection area.

### Statistical Analysis

All data were expressed as mean±SEM. Statistical analysis was carried out using a Student’s *t*-test fro comparison between two groups. A *P* value of <0.05 was considered significant.
